# Addendum: Immunoglobulin Gene Repertoire Diversification and Selection in the Stomach – From Gastritis to Gastric Lymphomas

**DOI:** 10.3389/fimmu.2014.00666

**Published:** 2015-01-05

**Authors:** Miri Michaeli, Hilla Tabibian-Keissar, Ginette Schiby, Gitit Shahaf, Yishai Pickman, Lena Hazanov, Kinneret Rosenblatt, Deborah K. Dunn-Walters, Iris Barshack, Ramit Mehr

**Affiliations:** ^1^The Mina and Everard Goodman Faculty of Life Sciences, Bar-Ilan University, Ramat Gan, Israel; ^2^Department of Pathology, Sheba Medical Center, Ramat Gan, Israel; ^3^Division of Immunology, Infection, and Inflammatory Diseases, King's College London School of Medicine, London, UK; ^4^Sackler Faculty of Medicine, Tel Aviv University, Tel Aviv, Israel

**Keywords:** B-cells, Ig gene, repertoire, somatic hypermutation, diversity

In Section “Diversity Analysis” of this article, there were some inaccuracies in the way diversity terms were referred to. Hence, we re-wrote the section, which should read as follows.

## Diversity Analysis

### Clones in samples can be regarded as species in habitats

In the case of lymphocyte clonal repertoire samples (e.g., those obtained from tissue biopsies), we treat each sample as a sample from a habitat, in which the “species” are the BCR or TCR clones found in the sample. Each of the clones may be composed of a number of different sequences. In TCR clones, all sequences are identical, but in BCR clones sequences from the same clone may be different due to somatic hypermutation, and one may choose to use only unique sequences found, or all sequences including multiplicate ones. The latter choice depends on whether identical sequences coming from different cells can be identified as such, or cannot be distinguished from sequence duplications caused by PCR amplification. If the former is true (as when using random barcoding in the PCR primers), then the number of sequences that come from different cells is known and can be used to estimate diversity. If not, then TCR diversity cannot be estimated, and BCR diversity can only be estimated based on the number of unique sequences and thus would usually only give a minimum estimate of the total diversity, as we have done in this study.

### Diversity indices

In order to quantify the diversity of clonal repertoires (such as antibody/BCR or TCR gene repertoires) in each experimental group, and later to be able to compare between two or more groups, we used diversity indices (such as the Species Richness, the Shannon entropy, or the Simpson concentration, which are indices of order 0, 1, and 2, respectively) (1). These indices take into account the number of species and (in indices of order >0) the frequency of members of a species (in our case, sequences) of each species (in our case, clone) in each habitat sample. In indices of order 0, diversity is defined simply as number of species (in our case, lymphocyte clones) in a sample. In order 1 indices, clone size (or frequency) are taken into account, as in the Shannon entropy when diversity is the sum of [–*p*_i_*ln(*p*_i_)], where *i* represents a species or clone and *p*_i_ represents its size (the number of members/sequences, see below). Order 2 indices attribute more weight to large clones, as in the Simpson concentration, which is the sum of *p*_i_^2^. In our studies, we used both order 1 and 2 diversity indices, i.e., the Shannon entropy and the Simpson concentration.

### Estimating the full repertoire from which each sample was taken

Considering the large numbers of sequences observed only once in each sample, it is likely that many rare clones in an individual’s original full repertoire were not detected. To account for the presence of unobserved “species” (clones), all diversity indices can be estimated for whole repertoires (rather than calculated for the sample) using the method described by Chao and Shen (2), which is based on a non-parametric estimation of diversity indices where there are undetected species. Chao and Shen’s approach utilizes the concept of sample coverage to adjust the diversity indices for clones that escaped sampling. The sample coverage is estimated from the proportion of species/sequences that are observed only once within a sample.

In our Ig gene repertoire studies, the abundance data (numbers of unique sequences) of antibody clones in each sample was used to estimate the mean, standard error, and 95% confidence intervals (CI) of the diversity index of choice (order 0, 1, and 2 indices) of the full repertoire from which each sample was taken (including unobserved clones). This was done using SPADE©, a program designed for diversity calculations (2).

### Diversity measures

From the estimated diversity indices for each sample or pool of samples, we have calculated the average alpha, beta, and gamma diversity measures for each group of samples (1). The average alpha diversity measure represents the average sample – or in our case, whole repertoire – diversity in each group of samples. In order to calculate the average alpha, we calculate the alpha diversity of each estimated repertoire using SPADE©(that is, $-\sum_{i=1}^{s}\left(p_{i}\times \ln p_{i}\right)$ for Shannon entropy, and $\sum_{i=1}^{s}\left(p_{i}^{2}\right)$ for Simpson concentration), and then average over all samples from the same group of samples. The gamma diversity measure represents the “global” repertoire diversity across all samples studied in each group of samples. It is calculated as the diversity of the pool containing all the repertoires estimated from all the samples in the same group of samples, also by SPADE©, using the same indices as for alpha.

Finally, the beta diversity measure, which represents the diversity component resulting from the variability between individual repertoires or samples, should in principle be calculated as the gamma of the group of samples divided by the average alpha of all samples in the group of samples. In order to compare between groups of samples, however, we needed to calculate CI for beta. This was done by calculating a beta measure per sample (the gamma of the group of samples divided by alpha of the sample) and then calculating the average, standard error, and CI of beta for each group of samples. Thus, comparisons can be made between the diversity measures calculated for different groups. One should keep in mind, however, that the comparisons of average alpha are based on averages of the estimates of alpha for all samples in a group, while the comparisons of gamma are based on the CI for the estimated diversity for each group. Since in all cases the 95% CI are given, if these intervals (e.g., of gamma) for two groups do not overlap, then the measures in question (e.g., gamma) of the two groups are significantly different with *p* < 0.05 under Student’s *t*-test (Figure 1).

**Figure 1:**
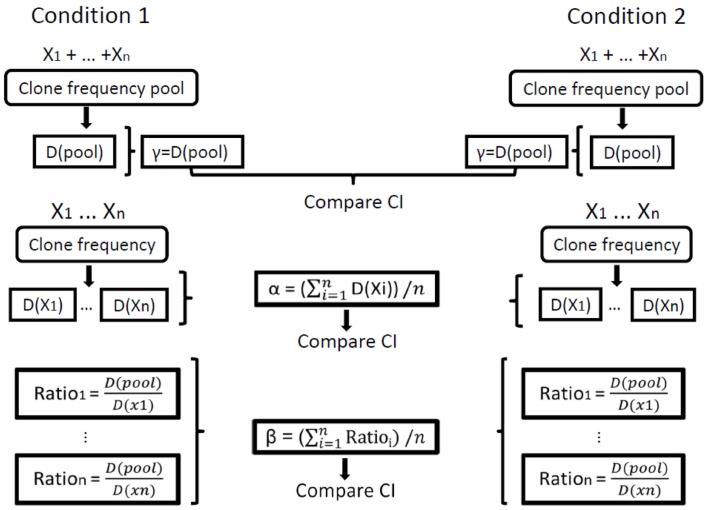
**Illustration of the calculation of repertoire diversity**. First, the diversity indices (Shannon entropy, Simpson concentration, etc.) are calculated for each sample. Next, the distribution measures (α, β, γ) are calculated for each condition using the individual samples. Finally, the confidence intervals (CI) of the distribution measures are compared between the populations/conditions. Xi represents the set of clone frequencies in sample *i*, that is, the set (for all samples) of the numbers of unique sequences in all clones in sample *i* out of the total number of unique sequences in the whole sample. *D* stands for Diversity, *D*(pool) is the calculation of γ (gamma) for a sample and *D*(Xi) is the calculation of α (alpha) for sample *i*. In our calculations of order 1, we used only the Shannon entropy for D, and for order 2 we used only the Simpson concentration for D.

In order to allow an intuitive comparison between the diversities of each of the groups, all the diversity measures can be expressed as their number equivalents (1), which reflect the number of equally sized clones needed to produce the given value of the diversity index.

## Similarity Analysis

In order to understand the sources for the differences in diversity between groups of samples, we used similarity analysis based on the Morisita similarity index (3). SPADE©(2) was used to calculate a similarity matrix, in which we measured each estimated individual repertoire’s similarity to all other estimated individual repertoires. A value close to 1 represents high similarity between two groups, and a value close to 0 represents low similarity. The average of similarity indices of individuals in a given group to those in another group represents the similarity index for the comparison between the two groups.

## Conflict of Interest Statement

The authors declare that the research was conducted in the absence of any commercial or financial relationships that could be construed as a potential conflict of interest.
